# Differential late-stage face processing in autism: a magnetoencephalographic study of fusiform gyrus activation

**DOI:** 10.1186/s12888-024-06400-z

**Published:** 2024-12-18

**Authors:** Darko Sarovic, Justin Schneiderman, Sebastian Lundström, Bushra Riaz, Elena Orekhova, Sheraz Khan, Christopher Gillberg

**Affiliations:** 1 Gillberg Neuropsychiatry Centre, Department of Psychiatry and Neurochemistry, Institute of Neuroscience and Physiology, Sahlgrenska Academy, University of Gothenburg, Gothenburg, Sweden; 2 Department of Radiology, Sahlgrenska University Hospital, Bruna Straket 11B, Gothenburg, 413 45 Sweden; 3 Athinoula A. Martinos Center for Biomedical Imaging, Boston, MA USA; 4 Department of Radiology, Massachusetts General Hospital, Harvard Medical School, Boston, MA USA; 5 Department of Clinical Neuroscience, Institute of Neuroscience and Physiology, University of Gothenburg, Gothenburg, Sweden; 6 Center for Neurocognitive Research (MEG Center), Moscow State University of Psychology and Education, Moscow, Russian Federation

**Keywords:** Autism, Magnetoencephalography, Biomarker, Fusiform face area, Pareidolia, Face processing

## Abstract

**Background:**

Autism is associated with alterations of social communication, such as during face-to-face interactions. This study aimed to probe face processing in autistics with normal IQ utilizing magnetoencephalography to examine event-related fields within the fusiform gyrus during face perception.

**Methods:**

A case–control cohort of 22 individuals diagnosed with autism and 20 age-matched controls (all male, age 29.3 ± 6.9 years) underwent magnetoencephalographic scanning during an active task while observing neutral faces, face-like pareidolic objects, and non-face objects. The fusiform face area was identified using a face localizer for each participant, and the cortical activation pattern was normalized onto an average brain for subsequent analysis.

**Results:**

Early post-stimulus activation amplitudes (before 100–200 ms) indicated differentiation between stimuli containing fundamental facial features and non-face objects in both groups. In contrast, later activation (400–550 ms) differentiated real faces from both pareidolic and non-face objects across both groups and faces from objects in controls but not in autistics. There was no effect of autistic-like traits.

**Conclusions:**

The absence of group differences in early activation suggest intact face detection in autistics possessing a normal IQ. Later activation captures a greater degree of the complexity and social information from actual faces. Although both groups distinguished faces from pareidolic and non-face objects, the control group exhibited a slightly heightened differentiation at this latency, indicating a potential disadvantage for autistics in real face processing. The subtle difference in late-stage face processing observed in autistic individuals may reflect specific cognitive mechanisms related to face perception in autism.

**Supplementary Information:**

The online version contains supplementary material available at 10.1186/s12888-024-06400-z.

## Background

Visual perception and social communication have an intricate relationship through the processing of faces. The successful visual interpretation of faces and the information they communicate are prerequisites for socially reciprocal face-to-face interactions. For this reason, it has been hypothesized that any deficit affecting face processing, from visual perception difficulties [1], to higher cognitive dysfunction [2] may underlie the social communication deficits in autism. Various aspects of face processing in autism (See reviews by [3, 4]; also [5]) have been investigated using both behavioral [6, 7] and neurophysiological methods [8–10].

A key neuroanatomical area in terms of face processing is the fusiform face area (FFA), which is sensitive to facial stimuli [11, 12]. Three time-windows, corresponding to various neurophysiological components of face perception in the FFA, have been described; each postulated to relate to a different stage in the processing of faces. The first component occurs around 130 ms (referred to as M130), and is an early component of bottom-up detection of faces based on low-level visual cues [13]. It is postulated to represent early and automatic recognition of negative valence in the facial expression [14, 15], or possibly act as a low fidelity face detector based on low-level cues that are statistically associated with faces [13, 16–18], possibly conveyed via the subcortical system [19, 20]. Some studies have found larger early amplitudes for faces compared to objects [21–23], while others have failed to replicate the finding [24, 25]. Some studies have reported that individuals with autism have a later peak latency [26, 27], but not those that included individuals with Asperger syndrome [28].

The second component occurs around 170 ms (referred to as M170) and can be considered the first stage of face identification [2, 16, 29–31]. However, results for individuals with autism are mixed (*no difference*: [7, 27], *later*: [2, 3, 10, 26, 32, 33]). A meta-analysis which mainly included children indicated a higher latency in individuals with ASD when including face-like objects and emotional faces, but no difference in amplitude [4]. Studies employing treatment interventions have shown reduced latencies [34]. Face-like objects, similar to faces, elicit higher amplitudes of this component [35–37], but this may depend on task instructions for individuals with autism [38].

The third, late component occurs between 250 and 550 ms (referred to as the late component), and relates to the differentiation between various emotional expressions (higher amplitude for emotional faces than neutral ones [14]), as well as between familiar and unfamiliar faces (in general higher for familiar faces, occasionally lower in some areas, [30, 39, 40]). This indicates an even higher level of processing, and recruitment of additional top-down cognitive mechanisms, including memory and social-emotional processing.

The fact that higher cognitive functions influence these components indicates that there are feedforward and feedback mechanisms involved in FFA activation. Several studies have investigated visual processing and found differences in autism with regard to phase amplitude coupling [41–44]. One study specifically investigated this coupling in the FFA [41] and found that typically developing individuals show an increase in coupling between alpha phase and gamma amplitude while viewing faces, with no such increase for individuals with autism.

Due to the general difficulty with integration of social information present in autism, the findings of aberrant face processing may be due to an interaction effect from the social information present in faces. By employing face-like objects, the social information content is decreased, allowing the processes specifically underlying the identification of faces to be investigated. Eye-tracking studies have found that toddlers with autism have an intact perception of face-like objects, which they perceive as faces as much as neurotypical young children do, although they seem to orient less to them [45, 46]. Conceptually, identification during visual processing occurs by either first-order relational properties, which denotes the general configuration and spatial relations among similar parts [47] – two eyes, a nose and a mouth from top to bottom define a face – or by the second-order relational properties, which relates to the distinctive relations within and between the individual features – such as the shape of the eyes or mouth, and the distances between them. face-like objects contain first-order relational properties similar to real faces but lack their complex social information and second-order relational properties. This may make them particularly useful in the study of face perception in autism which presents with a general difficulty with integration of social information.

While electroencephalography (EEG) has been instrumental in investigating face processing in autism, magnetoencephalography (MEG) has several advantages for studying neural dynamics. Unlike EEG, which measure electric potentials, MEG records magnetic fields generated by neural activity. Since magnetic fields are less distorted as they pass through the skull, MEG has a superior spatial resolution compared with EEG, making it particularly suitable for detecting activation within the FFA. Both methods are able to record neural activity with millisecond temporal resolution, allowing for averaging across stimulus presentations to estimate event-related potentials (for EEG) and fields (ERF; for MEG) in relation to specific stimuli.

The aim of the present study was to investigate the ERFs in the FFA, using MEG, in response to images that do and do not contain first- and second order relational properties of faces, in normal-IQ individuals with and without autism. Our hypothesis was that participants with autism have lower activation amplitudes in response to real faces during the late component, but with no difference in the early components, due to increased cognitive load in social processing.

## Methods

For a more detailed methodological description, see Supplementary Material 1.

### Participants

The study was approved by the Regional Ethical Board in Gothenburg (DNR: 552–14), and informed consent was gathered from all participants. The comparison individuals without autism (hereafter referred to as the control group) were recruited through flyers and the Gillberg Neuropsychiatry Centre website (GNC; www.gu.se/gnc). The individuals with an autism diagnosis were recruited from longitudinal studies currently ongoing at the GNC [48, 49] where they had been diagnosed using the Diagnostic and Statistical Manual of Mental Disorder – IV [50] and the International Classification of Diseases – 10 [51]. All patients were diagnosed by one of two experienced neuropsychiatrists and tested by one of two experienced neuropsychologists and re-diagnosed by the same clinical researchers (and still meeting criteria for autism spectrum disorder) at least five years after initial diagnosis. Although a full clinical examination was not performed on the control individuals, they were all seen by a clinical researcher (neuropsychologist), and both groups were interviewed by a medical doctor using a brief somatic and psychiatric checklist to check inclusion and exclusion criteria. The average Autism Spectrum Quotient [52] scores for the autism and control groups were 24.1 ± 9.1 and 11.4 ± 6.4 respectively (*p* < 0.00001, *d* = 1.64). The respective IQ scores were 109.8 ± 15.1 and 114.7 ± 11.6 (*p* = 0.25). Exclusion criteria were an intelligence quotient (IQ) below 80, co-occurring attention deficit/hyperactivity disorder for the autism group and any psychiatric diagnosis for the controls, current pharmacological therapy, having any implanted magnetic metal in the body, and vision problems that could not be corrected to normal (MEG-compatible glasses were used during recording for participants requiring correction).

We recruited and scanned 24 individuals with autism and 21 controls. Two patients and one control were excluded during analysis due to bad data quality (excessive noise, artifacts, or movement), such that 22 + 20 individuals were included in the analyses. All participants were adult males (age range 18.8 – 47.2 years, autism: 30.6 ± 7.1, control: 27.9 ± 6.6, *p* = 0.19). The groups were matched according to age and IQ. All subjects were recruited and scanned before analyses began, eliminating the risk of a biased subject sampling strategy [53].

### Data collection

IQ was tested in both groups with either the Wechsler Abbreviated Scale of Intelligence [54], or the Wechsler Adult Intelligence Scale-IV [55], depending on which study they were recruited from (since they used different tests). IQ-test scores were missing for two individuals with autism and one control. All participants completed the Autism Spectrum Quotient [52].

The participants underwent MEG scanning at the Swedish National Facility for Magnetoencephalography at the Karolinska Institute in Stockholm, Sweden. To allow for individualized anatomical localization of activity, T1-weighted brain magnetic resonance imaging (MRI) scans were obtained at three institutions using 3-Tesla MRI systems. A neuroradiologist reviewed each scan for image quality and possible pathologic findings. The recommended sequences for FreeSurfer ([56]; see http://surfer.nmr.mgh.harvard.edu) segmentation were used: MPRAGE for Siemens, FSPGR-BRAVO for GE, and TFE-SENSE for Philips. The scans were segmented with the FreeSurfer software [57, 58], using the watershed algorithm, to yield the gray and white matter surfaces, parcellations/segmentations of cortical and subcortical regions of interest (Desikan-Killiany atlas), and scalp surfaces (used for MEG-MRI data alignment).

### MEG experimental setup

The MEG recording was performed using a 306-channel whole-head MEG system (Elekta Neuromag TRIUX) within a two-layered magnetically shielded room, with active shielding engaged. For MEG-MRI co-registration, the head shape, three fiducial points and four head position indicator coils were digitized using a Fastrak system. We simultaneously recorded the electrocardiogram (ECG), and horizontal and vertical electrooculogram (EOG) for use in artifact removal. The MEG, ECG, and EOG signals were sampled at 1000 Hz.

**Fig. 1:**
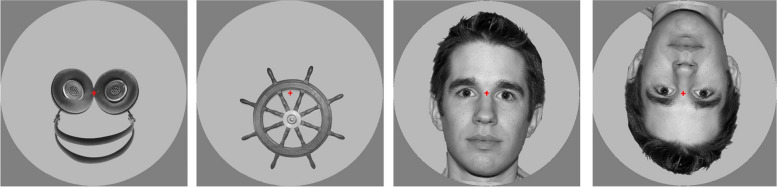
Examples of stimuli presented for OBJECTS paradigm. From left to right: pareidolic face-like object, non-face object, real face and inverted face

The face stimuli were obtained from the library NimStim Emotional Face Stimuli database [59]. Pareidolic face-like objects and objects were the same as those in a previous study ([35]; within which they performed a behavioral test to ensure face-like object stimuli were perceived as faces, and showed no difference in spatial frequency between the stimuli). See Fig. 1 for example stimuli. A red fixation cross was located at the center of the circle that contained the stimuli, such that it was also placed between the eyes of the face and face-like object stimuli, and the participants were instructed to maintain fixation on the cross. The experiment was made up of two paradigms: EMOTIONS, which consisted of faces expressing different emotions and was used to localize the face sensitive area in the fusiform gyrus (not for analyses in the present study); and OBJECTS, which consisted of inverted faces (control stimulus not used in the analyses), neutral faces, face-like objects, and objects (presented 80, 120, 240 and 120 times respectively). The participants were instructed to press a button whenever an inverted face was presented.

### MEG data preprocessing

To compensate for head movement, eliminate environmental magnetic noise, and the signal from active shielding, Elekta proprietary MaxFilter and temporal Signal Space Separation were used [60]. Both automatic (using thresholds of 5e-12 fT for magnetometers and 4e-10 fT for gradiometers) and manual (through visual inspection) exclusion of noisy channels and data segments (removing 1.0–1.2% of epochs) was performed. Principal Component Analysis (together with ECG- and EOG-channels) followed by Independent Component Analysis were used for identification and removal of biological artifacts. The data were epoched 200 ms before and 800 ms after stimulus onset, and baseline corrected using the 200 ms pre-stimulus interval. ERFs were generated by averaging the epochs for each stimulus type (except inverted faces) for the EMOTIONS and OBJECTS paradigms.

### Source localization pipeline

A grid consisting of 5121 dipoles was distributed across each hemisphere for the FreeSurfer-generated cortical surface. A single compartment boundary-element model was used for computation of the forward solution [61]. Co-registration of MEG-MRI data was performed for each individual against the digitized HPI coils, fiducials, and head shape. Alignment was first automatically performed, and then manually adjusted to minimize distances between digitized points and structural landmarks. For estimation of the MEG current distribution, we used the python based Minimum Norm Estimate [62, 63], which sets the dipole orientations as perpendicular to the cortex and constrains the neurophysiological signals to the cortex. The covariance matrix, estimated using the epoched data, was used to calculate the inverse operator. The inverse solution was applied using dynamic Statistical Parameter Maps (dSPM) to generate the source time courses. The bilateral labels corresponding to the medial brain and corpus callosum (“unknown”), were excluded from analysis.

### Fusiform face area localization: EMOTIONS-paradigm

Using the grand average for all upright face-stimuli in the EMOTIONS-paradigm, the area of maximal activity (dSPM value) between 140 and 200 ms post-stimulus within the label for the right FFA on the inferior temporal surface was identified, corresponding to each participant’s individual FFA. An outline of that area was created, and the vertices within that area were saved as a label. The first author (DS) created all the FFA label outlines, which were double checked by a neuroimaging researcher with experience in the field of visual processing to assure their plausibility.

### MEG data analysis: OBJECTS-paradigm

#### Event related fields

Activity within the FFA label was extracted from the source time course, corresponding to each participants’ source localized ERF. The ERFs were averaged across participants for each stimulus type (faces, face-like objects, objects), and group (autism and control). We analyzed both the continuous ERF (using cluster-based permutation testing), and the discrete amplitudes and latencies of the M130, M170, and late components. M170 was identified as the tallest peak in the 150–200 ms post-stimulus period. M130 was manually identified as the peak with inverse polarity occurring before M170. Their amplitudes were calculated as averages across the peak ± 10 ms. The presence of a late component was suggested by large clusters from the cluster-based permutation testing and was included as a dependent variable in the statistical testing. The late component amplitude was averaged across the 400–550 ms post-stimulus period, with the time window being identified post hoc from the permutation testing.

#### Statistical analyses

We structured our analyses into discrete (using analyses of variance (ANOVA) on component variables) and continuous (using cluster-based permutation testing on entire ERFs).

##### Analyses of variance

We used the statistical package SPSS (version 26; [64]) to perform mixed factor (for between-group analyses) and one-way repeated measures (for within-group analyses) ANOVA for each of the dependent variables: M130 peak latency, M130 amplitude, M170 peak latency, M170 amplitude, and late component amplitude.

An alpha-level of 5% was used to denote statistical significance. Sequential Holm-Bonferroni correction (HB; [65]) was performed within each family of observations for each main effect (since there were no significant interactions). Mixed ANOVA was performed with stimulus type as within-group, and diagnostic status as between-group independent variables. One-way ANOVA used stimulus type as the within-group variable. In the case of significant main effects, corrected Tukey post hoc tests were performed for pairwise comparisons where *p* < 0.05 was considered statistically significant.

##### Non-parametric cluster-based permutation testing

An in-house developed Python-based script for cluster-based permutation testing was used to compare groups and stimuli. The test compares the T-values across time series for randomized ERFs (group or stimulus type labels were randomized) from two comparison groups and extracts the largest T-value cluster (area under the curve for T-values above those corresponding to *p* < 0.05). The permutation tests were performed using 5´000 iterations, and comparison of the largest cluster of the actual data with the distribution of the largest permutated clusters yields a *p*-value that is corrected for multiple observations (within the time-series). HB-correction was applied within the families of between- and within-group analyses.

For each comparison and time-point, we calculate and present:

- the average ERF amplitudes with its 95% confidence interval
- an ERF subtraction plot with a 95% confidence interval
- significance across time (see Supplementary Material)
- absolute effect size (Cohen’s *d*) across times
- the proportion of individuals with a positive subtraction (for within-individual comparisons using stimulus types)
- the *p*-value for the comparison using cluster-based permutation test

#### Source localized activation

We also performed permutation testing on source localized activation patterns for between-group comparisons. A cluster-based permutation test (module within Minimum Norm Estimate-python; [66]) was used to compare conditions with 5´000 permutations and an F-distribution with a significance threshold of *p* = 0.001. We present those comparisons that were significant in the spatial cluster-based permutation testing.

## Results

We present the results for the discrete components, continuous ERFs, and source space activation below.

### Discrete components – comparisons of group and stimulus differences

**Fig. 2:**
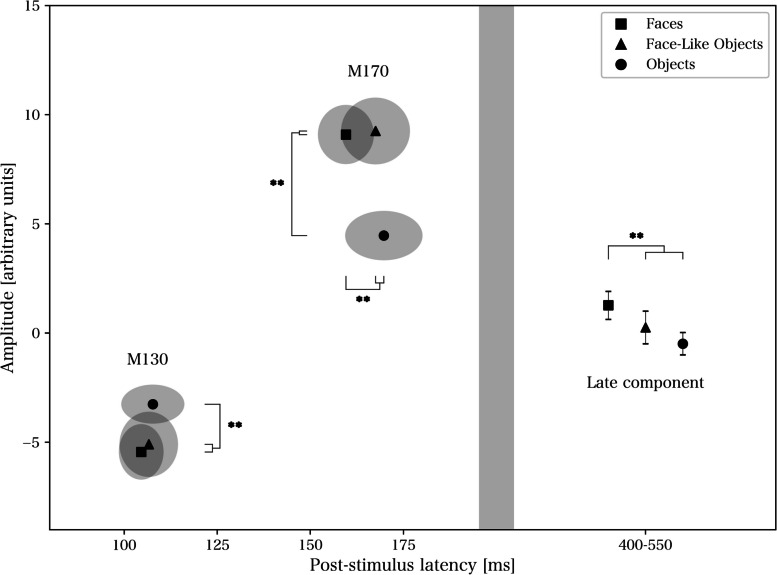
Across-group results of event-related fields within the right fusiform area. * and ** denote significant and Holm-Bonferroni-corrected significant differences for main effects from mixed analyses of variance

**Fig. 3:**
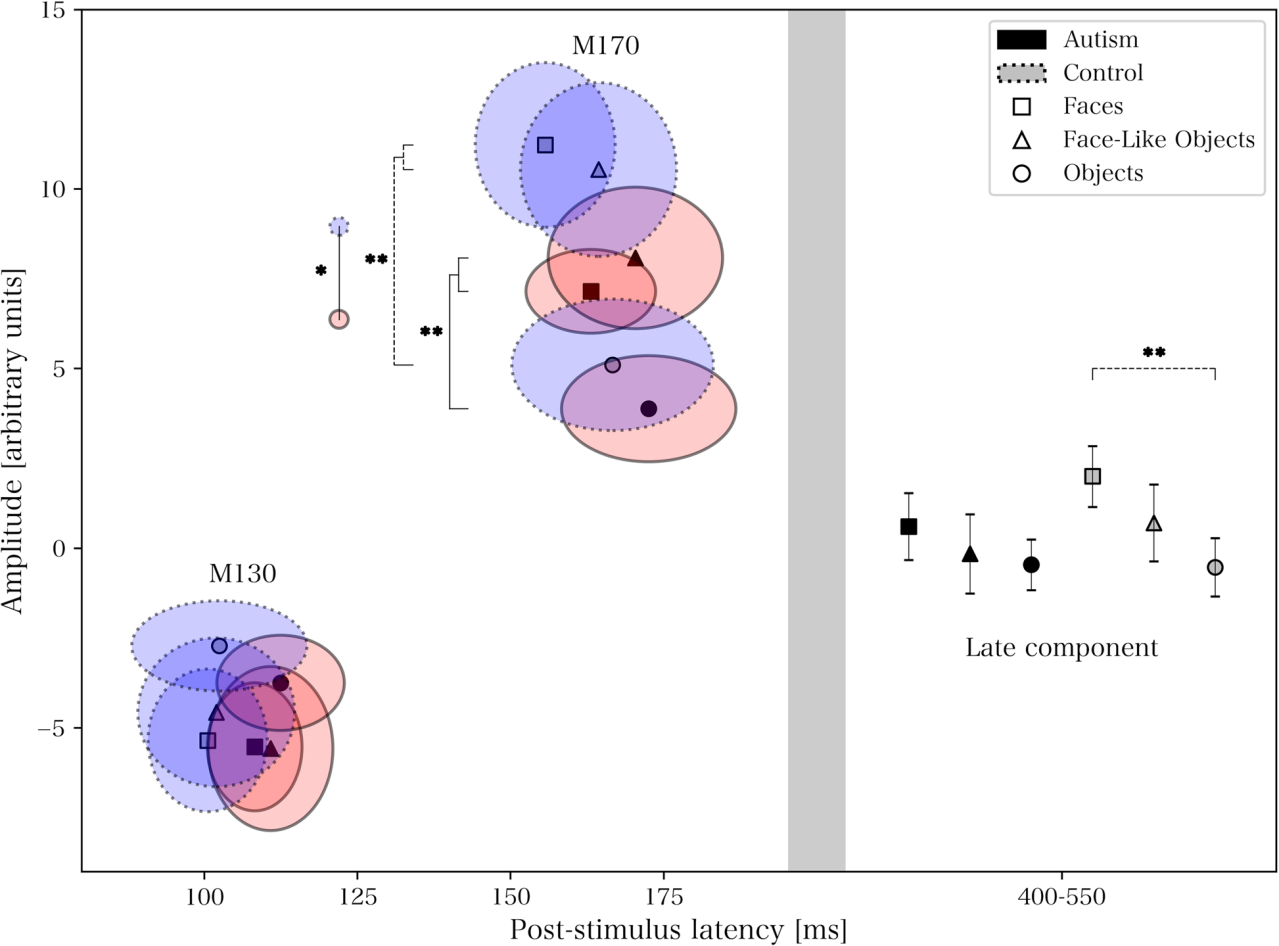
Within-group results of event-related fields within the right fusiform area. * and ** denote significant (*p* < .05) and Holm-Bonferroni-corrected significant differences (see Table 2 for *p*-value thresholds) for main effects from one-way analyses of variance

See Table 1 for descriptive statistics, and Figs. 2 and 3 for a presentation of the across- and within-group data and significant outcomes. For statistical analyses using mixed and one-way ANOVA, see Tables 2 and 3 respectively. Briefly, there were significant false discovery rate-corrected differences between stimulus types for M130 amplitude (objects lower amplitude than faces and face-like objects, across groups), M170 amplitude (objects lower amplitude than faces and face-like objects, both across and within groups), M170 latency (faces earlier than face-like objects and objects, across groups), and late component amplitude (faces higher amplitude than face-like objects and objects, across groups; faces higher amplitude than objects within the control group). There was also a between-group difference in the M170 amplitude (lower in the autism group), which was not significant after correction.

**Table 1:** Descriptive statistics for discrete components

		**Latency** **(ms; mean** ± **SD)**		**Amplitude** **(arbitrary units; mean** ± **SD)**		
**Group**	**Stimulus**	**M130**	**M170**	**M130**	**M170**	**Late**
Control	F	101 ± 21	156 ± 24	−5.35 ± 4.25	11.23 ± 4.90	2.00 ± 1.80
	FLO	102 ± 27	164 ± 27	−4.57 ± 4.43	10.54 ± 5.17	0.71 ± 2.28
	O	102 ± 31	167 ± 35	−2.71 ± 2.69	5.10 ± 3.91	−0.52 ± 1.74
Autism	F	108 ± 17	163 ± 24	−5.53 ± 4.03	7.15 ± 2.63	0.61 ± 2.10
	FLO	111 ± 23	170 ± 32	−5.57 ± 5.15	8.08 ± 4.45	−0.15 ± 2.49
	O	112 ± 23	173 ± 32	−3.74 ± 2.99	3.88 ± 3.33	−0.45 ± 1.59

Results presented as mean ± standard deviation (SD)

*ms* milliseconds, *F* Faces, *FLO* Face-Like Objects, *O* Objects

**Table 2:** Results of mixed ANOVA for discrete components: group x stimulus

Dependent variable	Outcome	Result	HB-α	Pairwise comparisons	
M130 amplitude	Interaction	*F*(1.74, 69.4) = .33, *p* = .69, η_p_^2^ = .008			
	Group	*F*(1, 40) = .51, *p* = .48, η_p_^2^ = .01	.05		
	Stimulus**	*F*(1.74, 69.4) = 7.78, *p* = .001, η_p_^2^ = .16, ε = .988	.0063	F-FLO	*p* = 1.0
				F-O*	*p* = .004
				FLO-O*	*p* = .001
M130 latency	Interaction	*F*(2, 80) = .13, *p* = .88, η_p_^2^ = .003			
	Group	*F*(1, 40) = 1.63, *p* = .21, η_p_^2^ = .04	.015		
	Stimulus	*F*(2, 39) = .92, *p* = .40, η_p_^2^ = .02, ε = .913	.017		
M170 amplitude	Interaction	*F*(2, 39) = 2.88, *p* = .07, η_p_^2^ = .13			
	Group*	*F*(1, 40) = 6.52, *p* = .02, η_p_^2^ = .14	.0083		
	Stimulus**	*F*(2, 39) = 42.12, *p* < .001, η_p_^2^ = .68, ε = 0.988	.005	F-FLO	*p* = 1.0
				F-O*	*p* < .001
				FLO-O*	*p* < .001
M170 latency	Interaction	*F*(2, 39) = .04, *p* = .96, η_p_^2^ = .002			
	Group	*F*(1, 40) = .60, *p* = .44, η_p_^2^ = .02	.025		
	Stimulus**	*F*(2, 39) = 6.40, *p* = .004, η_p_^2^ = .25, ε = 0.840	.007	F-FLO*	*p* = .03
				F-O*	*p* = .008
				FLO-O	*p* = 1.0
Late component amplitude	Interaction	*F*(2, 39) = 2.93, *p* = .07, η_p_^2^ = .13			
	Group	*F*(1, 40) = 1.94, *p* = .17, η_p_^2^ = .05	.01		
	Stimulus**	*F*(2, 39) = 18.22, *p* < .001, η_p_^2^ = .48, ε = 0.989	.0056	F-FLO*	*p* = .003
				F-O*	*p* < .001
				FLO-O	*p* = .057

*ANOVA* Analyses of Variance, *F *Faces, *FLO *Face-Like Objects, *O* Objects

^*^p < .05 (pairwise comparisons are Tukey corrected)

**HB-α: Holm-Bonferroni corrected p-threshold

**Table 3:** Results of one-way ANOVA for discrete components: within-group comparisons of stimulus

Group	Dependent variable	Result	HB-α	Pairwise comparisons	
Control	M130 amplitude	*F*(2, 57) = 2.44, *p* = .1, η^2^ = .08	.007		
	M130 latency	*F*(2, 57) = .03, *p* = .97, η^2^ = .001	.05		
	M170 amplitude**	*F*(2, 57) = 10.22, *p* < .001, η^2^ = .26	.005	F-FLO	*p* = .89
				F-O* (F higher)	*p* < .001
				FLO-O* (FLO higher)	*p* = .002
	M170 latency	*F*(2, 57) = .79, *p* = .46, η^2^ = .03	.013		
	Late amplitude**	*F*(2, 57) = 8.35, *p* < .001, η^2^ = .23	.0063	F-FLO	*p* = .1
				F-O* (F higher)	*p* < .001
				FLO-O	*p* = .12
Autism	M130 amplitude	*F*(2, 63) = 1.39, *p* = .26, η^2^ = .04	.01		
	M130 latency	*F*(2, 63) = .22, *p* = .81, η^2^ = .007	.03		
	M170 amplitude**	*F*(2, 63) = 8.49, *p* < .001, η^2^ = .21	.0056	F-FLO	*p* = .66
				F-O* (F higher)	*p* = .009
				FLO-O* (FLO higher)	*p* < .001
	M170 latency	*F*(2, 63) = .62, *p* = .54, η^2^ = .02	.02		
	Late amplitude	*F*(2, 63) = 1.51, *p* = 23., η^2^ = .05	.008		

*ANOVA* Analyses of Variance, *F* Faces, *FLO *Face-Like Objects, *O *Objects

^*^p < .05 (pairwise comparisons are Tukey corrected)

**HB-α: Holm-Bonferroni corrected p-threshold

We also performed exploratory analyses correlating component outcomes and behavioral data to address individual variations. Table 4 presents the results for each component and stimulus compared with AQ and full-scale IQ, including its subdomains (verbal, performance, working memory, speed). After correction for multiple observations, there were no significant correlations.

**Table 4:** Correlations between dependent variables and behavioral measures

Component	Stimulus	AQ	IQ	VIQ	PIQ	WMIQ	SIQ
M130 amplitude	F	*r* = .006 *p* = .97	*r* = -.21 *p* = .19	*r* = -.17 *p* = .31	*r* = *-.22* *p* = .18	*r* = -.17 *p* = .29	*r* = -.21 *p* = .19
	FLO	*r* = -.15 *p* = .34	*r* = -.04 *p* = .80	*r* = -.06 *p* = .71	*r* = -.06 *p* = .70	*r* = .13 *p* = .44	*r* = .04 *p* = .82
	O	*r* = -.29 *p* = .07	*r* = -.01 *p* = .94	*r* = -.12 *p* = .46	*r* = .009 *p* = .96	*r* = .14 *p* = .38	*r* = .06 *p* = .73
M130 latency	F	*r* = .14 *p* = .38	*r* = *-.02* *p* = .92	*r* = .17 *p* = .29	*r* = -.05 *p* = .75	*r* = -.15 *p* = .34	*r* = -.08 *p* = .65
	FLO	*r* = .023 *p* = .86	*r* = -.07 *p* = .67	*r* = .08 *p* = .64	*r* = -.11 *p* = .52	*r* = -.08 *p* = .61	*r* = -.13 *p* = .41
	O	*r* = .10 *p* = .55	*r* = *-.14* *p* = .41	*r* = .06 *p* = .71	*r* = -.16 *p* = .33	*r* = -.21 *p* = 19	*r* = -.18 *p* = .26
M170 amplitude	F	*r* = *-.32* *p* = .045*	*r* = *.25* *p* = .12	*r* = .13 *p* = .42	*r* = .02 *p* = .90	*r* = .37 *p* = .018*	*r* = .22 *p* = .18
	FLO	*r* = -.16 *p* = .33	*r* = .34 *p* = .031*	*r* = .16 *p* = .33	*r* = .39 *p* = .013*	*r* = .16 *p* = .32	*r* = .18 *p* = .28
	O	*r* = -.21 *p* = .19	*r* = .14 *p* = .40	*r* = -.04 *p* = .81	*r* = .03 *p* = .87	*r* = .20 *p* = .23	*r* = .26 *p* = .10
M170 latency	F	*r* = .07 *p* = .64	*r* = *-.10* *p* = .56	*r* = *.14* *p* = .40	*r* = -.14 *p* = .38	*r* = -.15 *p* = .35	*r* = -.12 *p* = .46
	FLO	*r* = .04 *p* = .80	*r* = .10 *p* = .54	*r* = .16 *p* = .32	*r* = .08 *p* = .63	*r* = -.05 *p* = .75	*r* = .12 *p* = .45
	O	*r* = .12 *p* = .47	*r* = *-.05* *p* = .78	*r* = *.10* *p* = .53	*r* = -.12 *p* = .48	*r* = -.11 *p* = .52	*r* = .01 *p* = .94
Late amplitude	F	*r* = -.27 *p* = .09	*r* = .02 *p* = .91	*r* = .06 *p* = .70	*r* = -.03 *p* = .85	*r* = .10 *p* = .55	*r* = -.13 *p* = .42
	FLO	*r* = -.14 *p* = .39	*r* = -.05 *p* = .76	*r* = -.03 *p* = .85	*r* = .07 *p* = .69	*r* = -.07 *p* = .68	*r* = -.23 *p* = .16
	O	*r* = -.06 *p* = .71	*r* = -.08 *p* = .64	*r* = -.05 *p* = .78	*r* = -.04 *p* = .81	*r* = -.09 *p* = .57	*r* = -.02 *p* = .89

*F* Faces, *FLO* Face-Like Objects, *O* Objects

^*^ *p* < .05, before FDR-correction

### Continuous ERF – comparing group and stimulus type

**Fig. 4:**
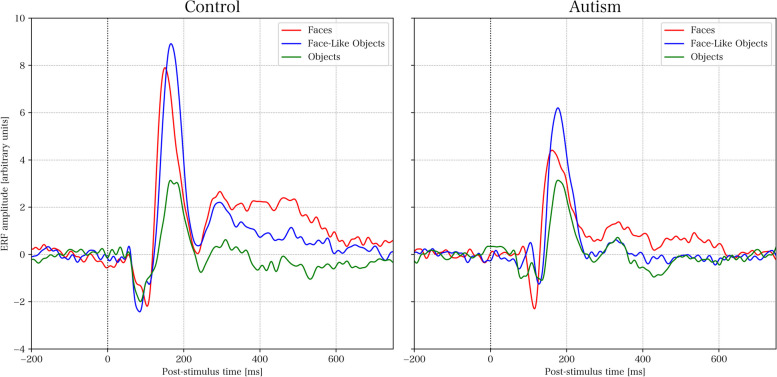
Source space activations within the right fusiform face area averaged for each group and stimulus

**Fig. 5:**
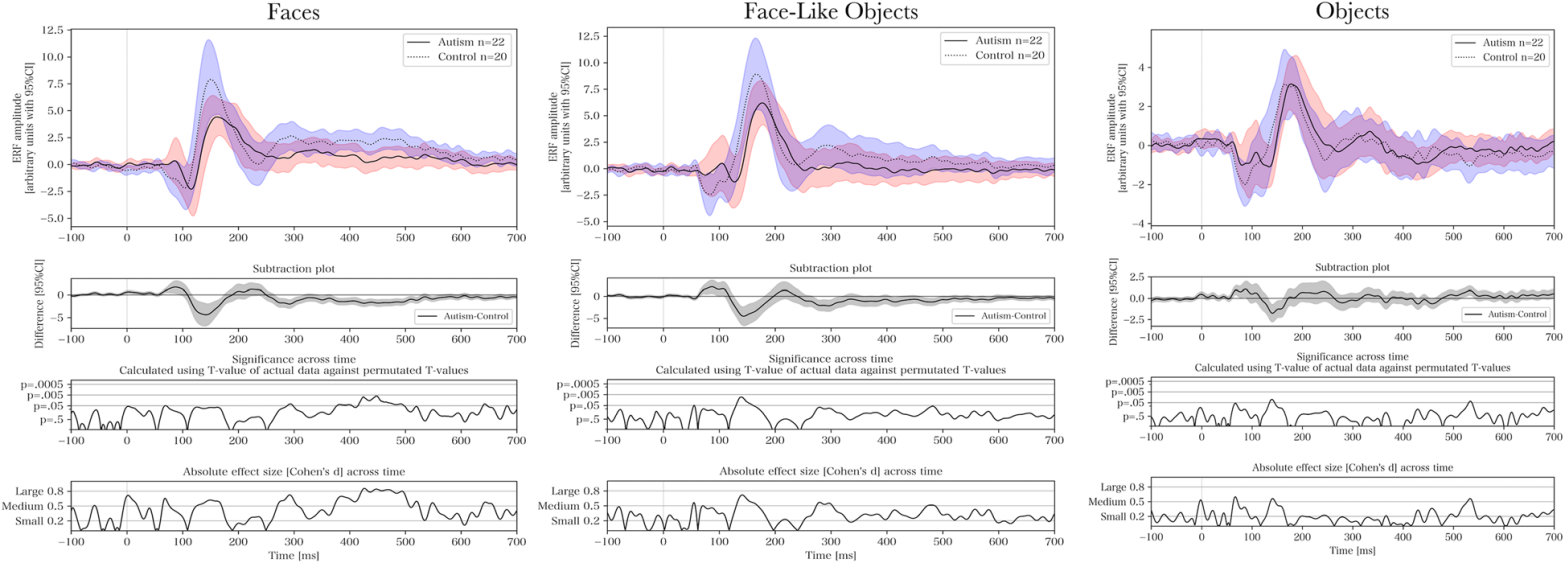
Between-group pairwise group comparison for each stimulus type. Cluster-based permutation test for event-related fields with 5000 permutations when comparing autism and control for faces (*p* = .10), face-like objects (*p* = .44), and objects (*p* = .60). From top to bottom, the subplots present, for each time-point, the averaged event-related field (ERF) activation, a stimulus-wise subtraction for the averaged ERFs, and the permutation-based T-values and effect sizes

**Fig. 6:**
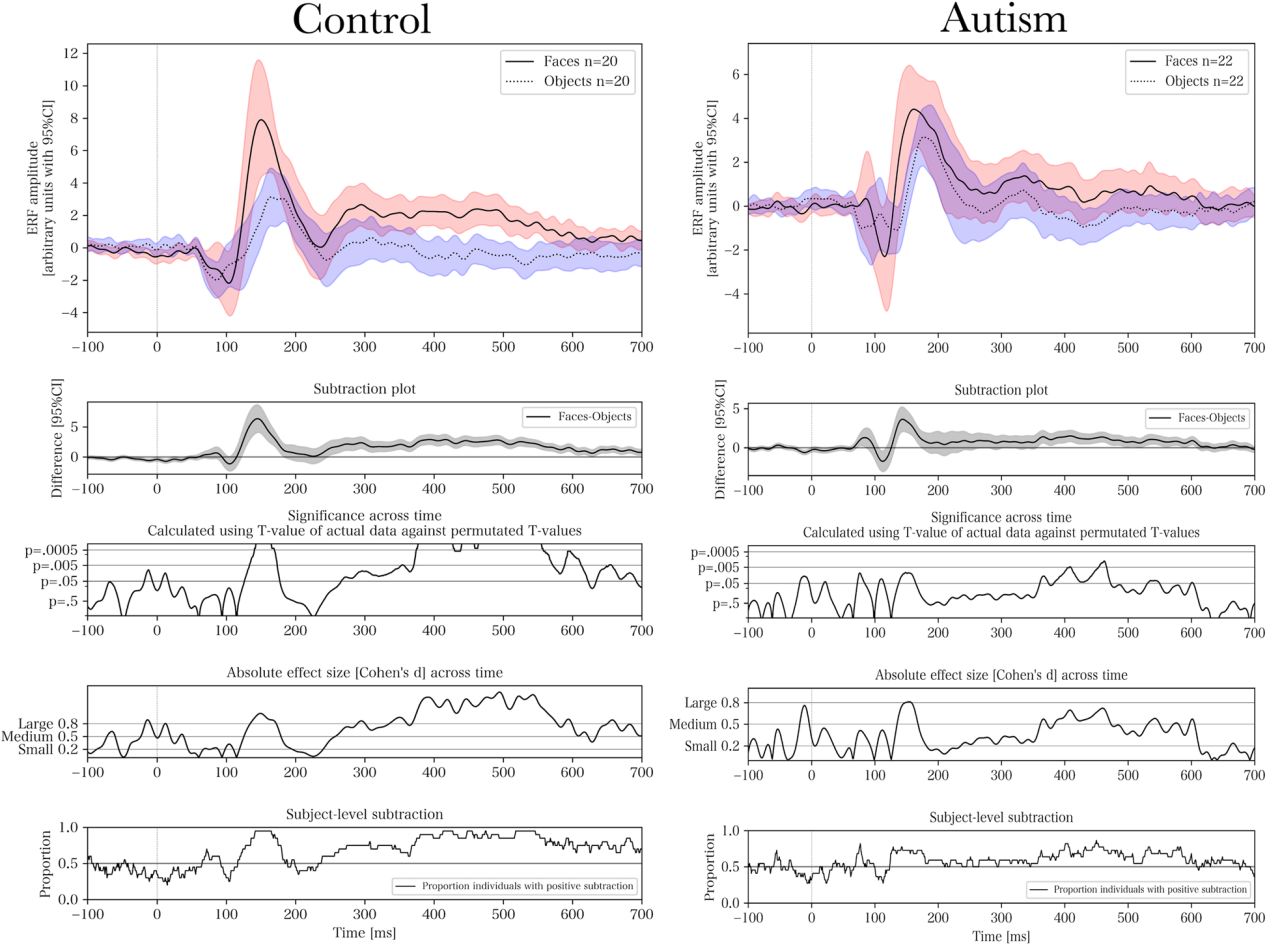
Within-group pairwise comparisons between faces and objects. Cluster-based permutation test for event-related fields with 5000 permutations when comparing faces and objects for control (*p* < .0002) and autism (*p* = .067). From top to bottom, the subplots present, for each time-point, the averaged event-related field (ERF) activation, a stimulus-wise subtraction for the averaged ERFs, permutation-based T-values and effect sizes, and the proportion of individuals with positive within-subject subtractions

**Fig. 7:**
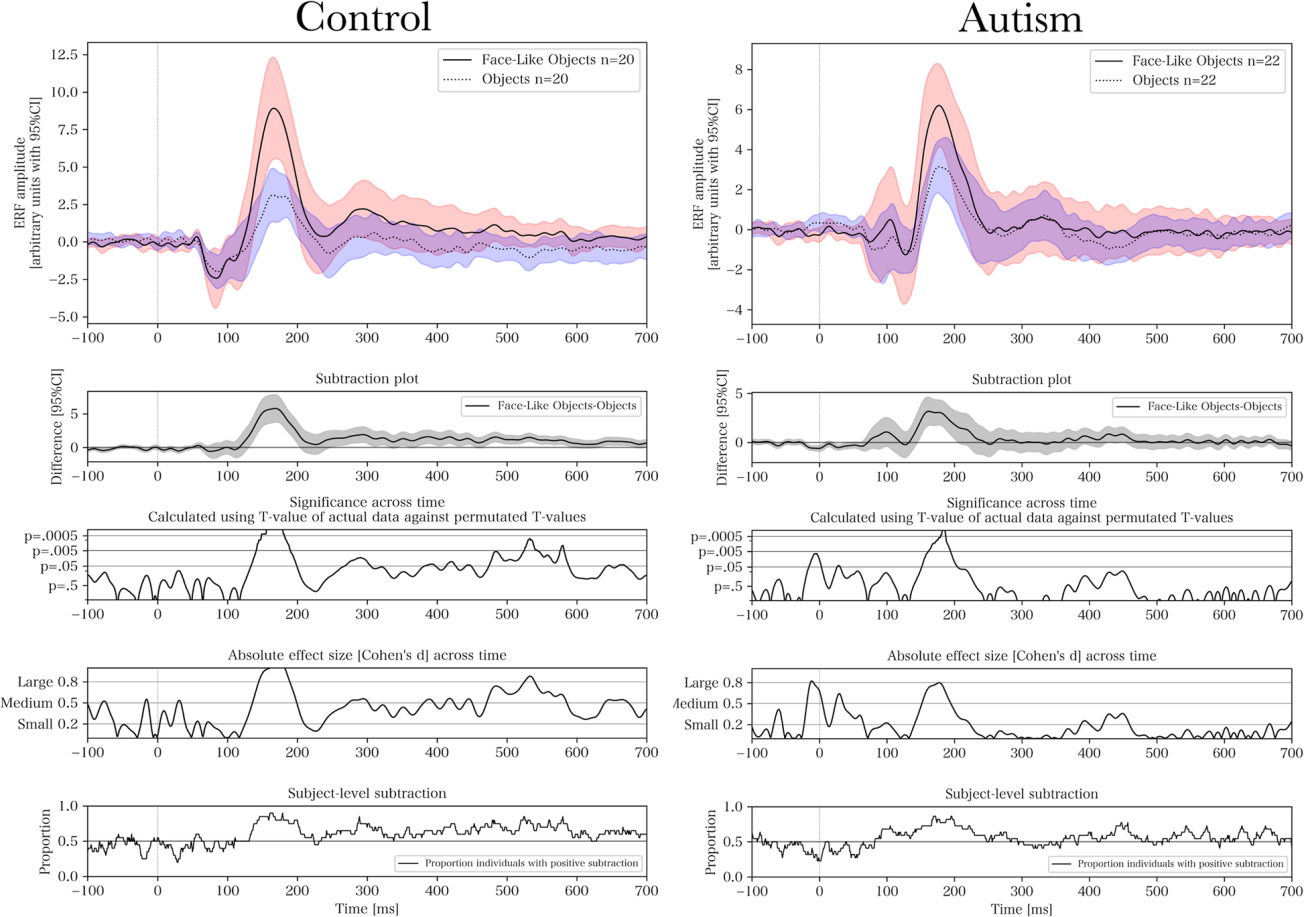
Within-group pairwise comparisons between face-like objects and objects. Cluster-based permutation test for event-related fields with 5000 permutations when comparing face-like objects and objects for control (*p* = .027) and autism (*p* = .053). From top to bottom, the subplots present, for each time-point, the averaged event-related field (ERF) activation, a stimulus-wise subtraction for the averaged ERFs, permutation-based T-values and effect sizes, and the proportion of individuals with positive within-subject subtractions

**Fig. 8:**
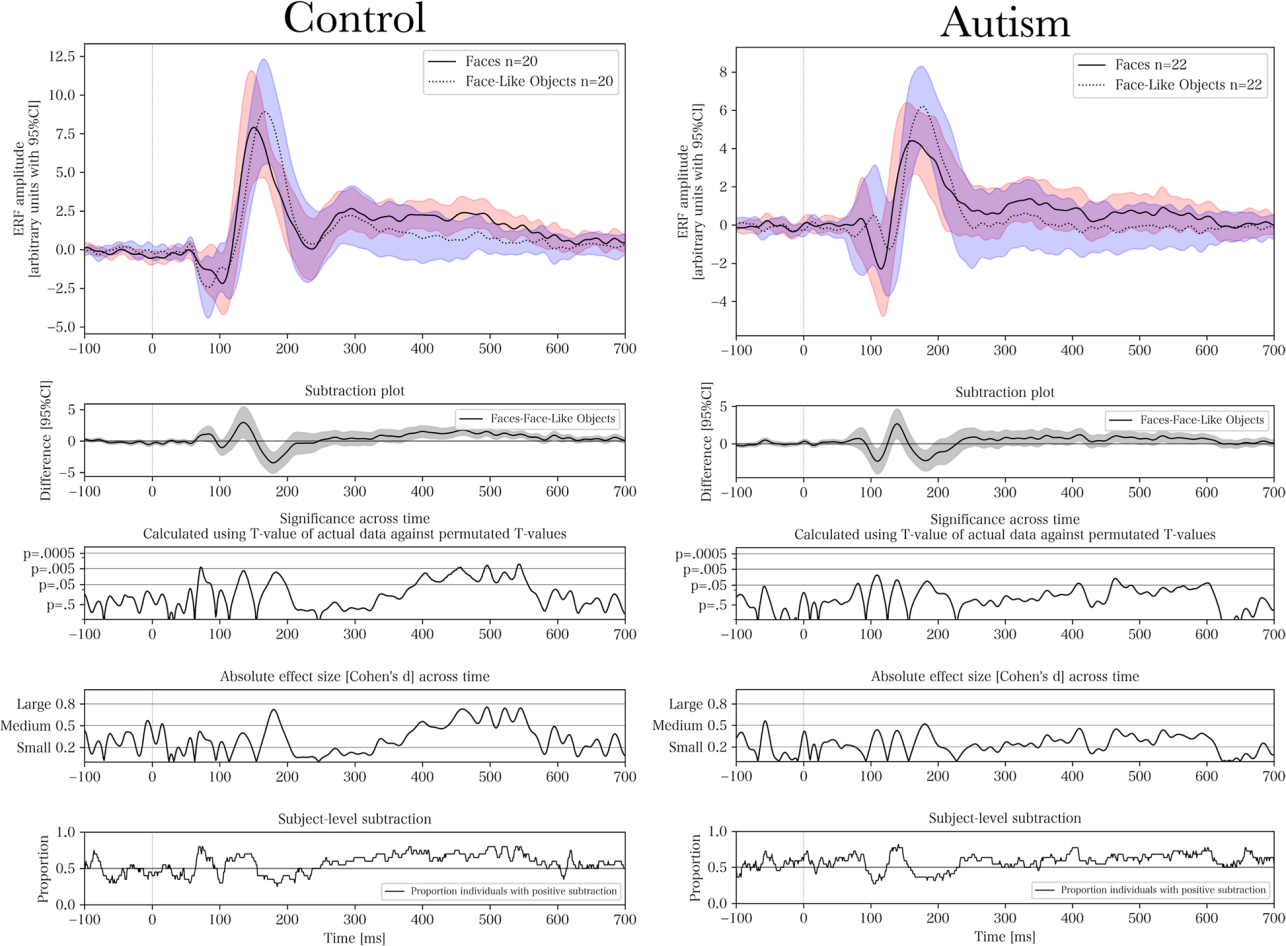
Within-group pairwise comparisons between faces and face-like objects. Cluster-based permutation test for event-related fields with 5000 permutations when comparing faces and face-like objects for control (*p* = .02) and autism (*p* = .63). From top to bottom, the subplots present, for each time-point, the averaged event-related field (ERF) activation, a stimulus-wise subtraction for the averaged ERFs, permutation-based T-values and effect sizes, and the proportion of individuals with positive within-subject subtractions

Figure 4 presents the ERFs for each group and stimulus type. For pairwise comparisons of groups and stimuli using permutation testing, see Figs. 5, 6, 7 and 8 respectively. The results of the permutation tests are presented in Table 5. Briefly, there were no between-group differences when comparing individual stimuli. There were pair-wise differences between all the stimuli across groups (see Figures S1-S3 in Supplementary Material). There was a difference between faces and objects within the control group (see left panel of Fig. 6; and between faces and face-like objects, and between face-like objects and objects before correction for multiple comparisons). There were no differences within the autism group.

**Table 5:** Results of permutation testing of event related fields

Variable	Data used	Comparison	Outcome	HB-α
Stimulus	All individuals	F-FLO** (F higher at late component)	*p* = .01	.0125
		F-O** (F higher at M170 and late component)	*p* = .0001	.0083
		FLO-O** (FLO higher at M170)	*p* = .0082	.01
Between-group	F	Autism-Control	*p* = .1	.017
	FLO	Autism-Control	*p* = .44	.025
	O	Autism-Control	*p* = .6	.05
Within-group	Autism	F-FLO	*p* = .63	.05
		F-O	*p* = .067	.025
		FLO-O	*p* = .053	.017
	Control	F-FLO* (F higher at late component)	*p* = .02	.01
		F-O** (F higher at M170 and late component)	*p* < .0002	.0083
		FLO-O* (FLO higher at M170)	*p* = .027	.0125

*F* Faces, *FLO *Face-Like Objects, *O *Objects

^*^ *p* < .05

**HB-α: Holm-Bonferroni corrected p-threshold

### Source localized activation

**Fig. 9:**
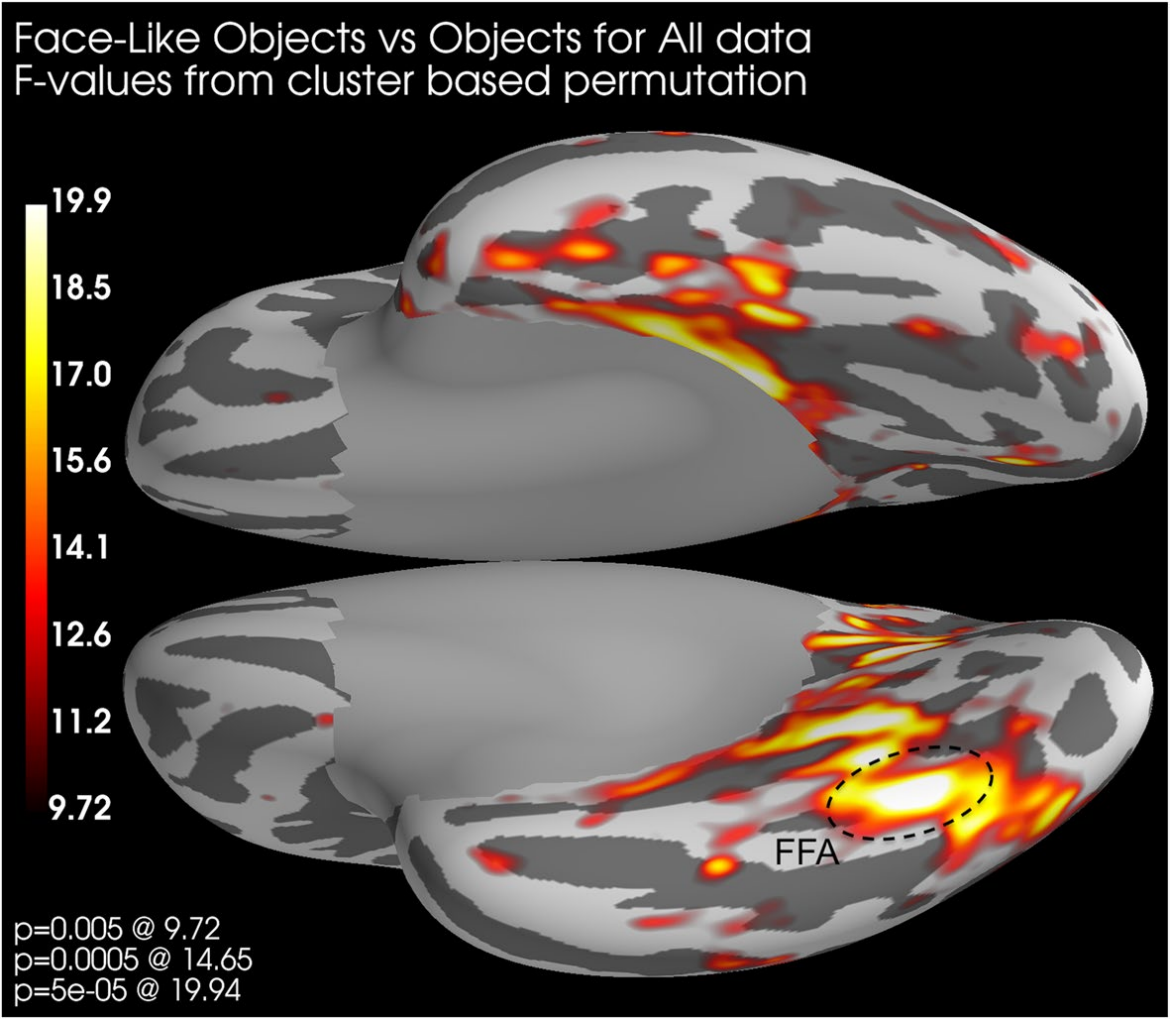
Source space distribution of *F*-values around 150–170 ms from cluster-based permutation when comparing face-like objects and objects across all participants

**Fig. 10:**
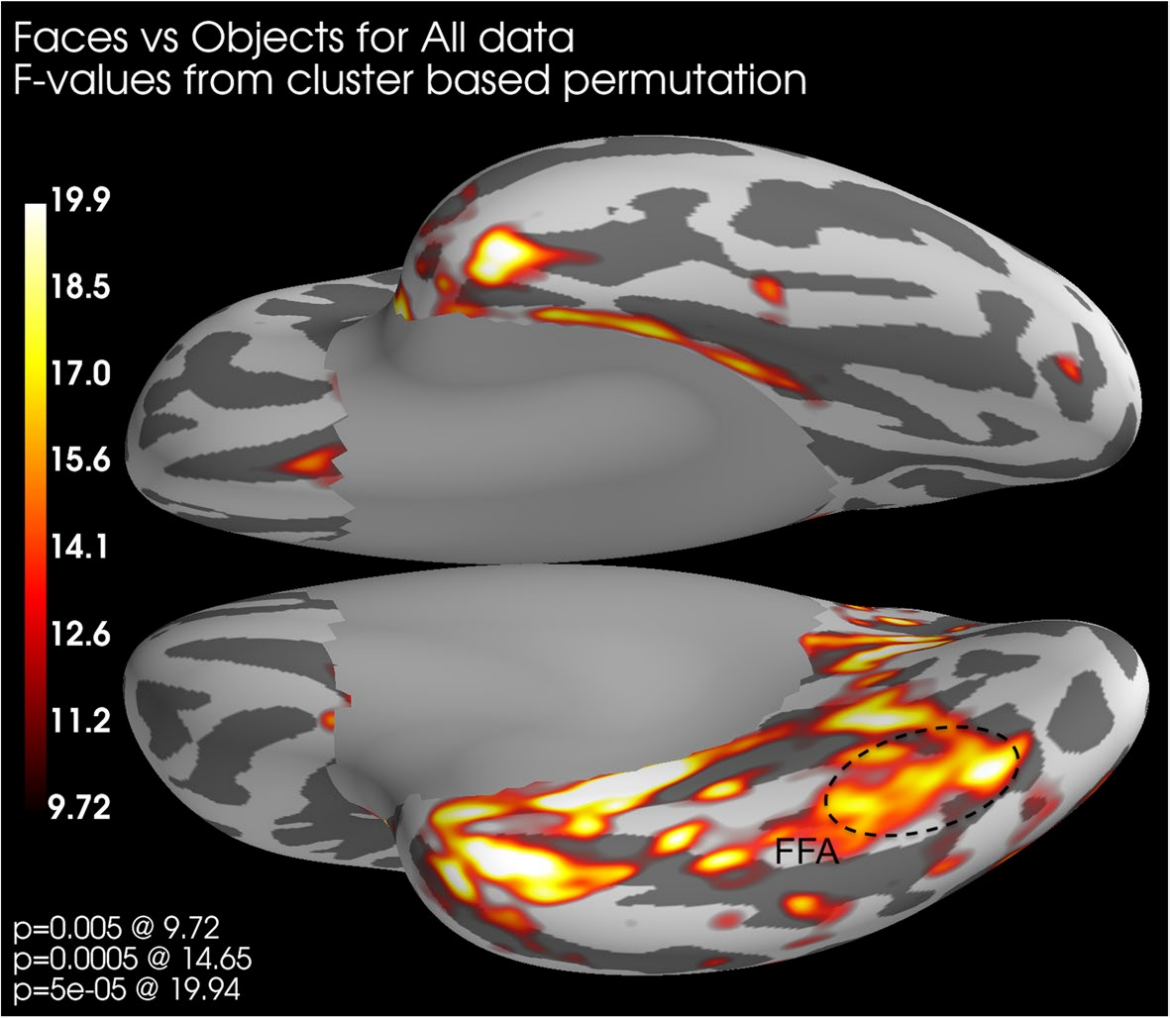
Source space distribution of *F*-values around 130–150 ms from cluster-based permutation when comparing faces and objects across all participants

Cluster-based permutation for the source space activation for the comparison between face-like objects and objects indicated a spatial cluster (high *F*-values) in the area corresponding to the right FFA (Fig. 9) between 150 and 170 ms. For the comparison between faces and objects, there was also a spatial cluster in the FFA, with additional clusters in the anterior inferior aspect of the temporal lobe (Fig. 10) between 130 and 150 ms.

## Discussion

The aim of this study was to investigate the neurophysiological signal within the FFA of individuals with autism and neurotypical controls when viewing faces, objects containing face-like features but devoid of social information, and objects. The findings are discussed for component and stimulus results on the one hand, and group differences on the other.

### Components and relation to differentiation of first- and second-order relational properties

We found no difference between groups in latency at the M130 component. Previous studies using case–control cohorts have yielded mixed results pertaining to differences in the peak latency of M130. Wong et al. [27] and O’Connor et al. [26] found a later peak latency in individuals with autism, while O’Connor et al. [28] did not. The latter consisted of normal-IQ individuals with autism, as did our sample. This may indicate that confounding factors, such as cognitive disability, underlie the delayed timing of M130. This has also been found with regard to later components (P300; [67, 68]).

We did, however find, as expected, a lower amplitude for objects at both the M130 and M170 in both groups. M170 has consistently been reported to show an earlier peak latency and higher amplitude for faces than objects [2, 10, 17, 26, 69–72]. While Churches et al. [36], found an intermediate amplitude for face-like objects, we found no significant difference in amplitudes for faces and face-like objects.

Churches et al. [36] only included a time-window until 300 ms, but presented a positive deflection for faces after 250 ms, hinting at a late component for faces that may differ from that of face-like objects and objects. Similarly, Wardle et al. [73] found increased classification accuracy when comparing faces with face-like objects and objects at the late component. We also found an increased amplitude for faces compared to the other stimuli across groups, and this is interpreted as differentiation of real faces from non-face stimuli, including face-like objects. This in turn implies that the late component may be sensitive to second-order relational properties, or to the social content of the stimulus.

With increasing time from stimulus presentation, there is increased time to recruit other neural networks and incorporate higher cognitive functions. This is exemplified by the pattern of findings at the three components (M130, M170 and the late component). Previous studies have suggested that M130 is involved in unconscious, fast and negative valence appraisal [14, 15] and we did not employ fearful faces. We did, however, find a lower M130 amplitude for objects than both faces and face-like objects. This may be because our stimuli that contained first-order relational properties of faces were processed as such, which are socially salient stimuli. The results regarding the differentiation of faces from non-face objects at M130 are inconsistent [13, 17, 24]. M170 has been suggested to represent the first stage of perception of a stimulus as being a face [2, 16, 29, 30]. The fact that the M170 in our analyses differentiates face-like stimuli from non-face objects corroborates this as an unspecific component of face-detection, but the detection of a face may occur as early as the M130 (at least as suggested in our study, and that of Hadjikhani et al. [35]). The late component (> 250 ms) has been found to identify familiar faces and emotional expressions [14, 30, 39, 40], suggesting recruitment of higher cognitive mechanisms, including memory and social cognition. Although we did not employ familiar faces or emotional expressions in our analysis, the fact that the late component differentiated faces from both objects and face-like objects indicates that the late component is sensitive to second-order relational properties, perhaps due to increased social information.

### Group differences

There were no significant differences following ANOVA and permutation testing when comparing how the groups process each stimulus type. Wong et al. [27] found an earlier M170 latency for the control group for all stimuli. Even though we had twice the sample size, we did not find a group difference in M170 latency, meaning that lack of power does not explain the lack of significant difference. The difference may be due to the fact that Wong et al. [27] included children aged 6–10, while we had an adult sample. A systematic review investigating the effect of age on M170 latency may shed light on this difference.

Previous studies [74–76] have found a lower M170 amplitude in the autism group for faces and face-like objects compared with a control group. We found a trend toward a group difference for M170 amplitude, but it was not significant following false discovery rate-correction. A larger sample size may have identified a significant group difference for amplitude. There were identical amplitudes for objects between the groups, implying that this the slightly lower amplitude at M170 for the autism group is not explained by extraneous factors related to scanning such as an increased distance to the sensors (due to positioning or differences in head size).

Even though there were no significant between-group differences for individual stimuli using permutation testing, there were within-group differences. The largest within-group difference occurred in the late component. The control group had a significantly higher amplitude for faces compared with objects (also found by [73]), with no differences between stimuli in the autism group. However, the lack of differentiation at the later component, which is more reliant on higher cognitive modulation, could indicate that individuals with autism may have difficulty with top-down integration of social information [2]. We found no indication that the amplitude of the late component relates to the degree of autistic-like traits, implying that the difference observed within that specific time window might be a feature of the clinical status rather than the behavioral autistic phenotype. It has been proposed that the presence of risk factors (such as pregnancy related complications) affecting higher cognitive integration induces a clinical sampling bias [77]. Because risk factors are mostly non-specific for autism and may contribute to a clinical sampling bias for neurodevelopmental disorders in general, this finding may be identified in other conditions as well. This finding needs to be replicated in other studies, but it would also be interesting to look at this in samples with other neurodevelopmental conditions, such as ADHD, to investigate its specificity.

Previous studies that identified alterations in M130 included samples with lower IQ [26], whereas normal-IQ samples, including ours, do not show such alterations [28]. This suggests an interaction with cognitive ability, and the possibility that some findings that have been attributed to autism may be due to lower intelligence. This supports the notion of stratification according to cognitive ability for autism cohorts.

### Limitations and strengths

A major strength is that our patient sample was very well phenotyped; all patients received a diagnosis of autism on at least two separate occasions and have been identified and followed up through longitudinal cohorts. Another strength is that the groups were age- and IQ-matched.

Additional strengths compared with some of the previous literature is that we employed MEG – although it has a different spatial sensitivity profile from EEG, it has a higher spatial resolution – and extracted the ERF from a common source space activation rather than from the sensor space – yielding a higher spatial specificity for the FFA and limiting noise due to variability from electrode placement and FFA location [78]. On the other hand, there are limitations to the use of source space activations which are based on the assumptions of the model and analysis method used.

As with most other MEG studies on patient populations, ours had a small sample. Although the high temporal and spatial resolution of MEG means there is adequate statistical power for detecting group and stimulus differences, one cannot exclude the possibility that the findings presented are idiosyncratic for the current sample and lack generalizability. A few comparisons showed statistically significant differences but failed to remain significant after false discovery rate-correction, indicating that we might not have had adequate power for those comparisons. Although our focus was to investigate face processing in a normal-IQ sample, the exclusion of individuals with other neuropsychiatric disorders, such as ADHD and intellectual disability, limits the generalizability of our findings. Future studies should aim to include both larger and more clinically diverse samples, to allow for subgroup analyses and interaction effects to be elucidated. Another substantial limitation is that we only included adult males (which was due to convenience as we had access to two exclusively male longitudinal samples) and replication studies on females and children are warranted. Notably, normal-IQ females have significantly more pronounced behavioral camouflaging [79, 80], and it is possible that females might show different neural dynamics. Future research will need to address this and directly compare males and females.

Even though we controlled for image size and luminance, there are several more low-level visual properties that are hard to account for. We used a categorical design (comparison between complex visual stimuli containing both low- and high-level differences) which doesn’t allow us to draw conclusions about stimulus specificity; the differences we observed may be due to uncontrolled low-level stimulus properties [81]. The spatial frequency did not differ between our stimuli [35] and the inclusion of face-like objects at least controls for the presence of first- and second-order relational properties. However, some of the non-faces filled less of the visual field which could be negatively affecting activation estimates. However, the facial features for the face-like objects and the center of field of view for objects were located in similar positions as the faces stimuli, aiming to control for face detection and since the FFA activation relates more to detection of facial features than direct retinal stimulation, this might be less of an issue in the FFA than the primary visual cortex.

### Methodological discussion

Studies indicate that individuals respond neurophysiologically to face-like objects as to faces when primed for faces [37, 82], indicating a degree of top-down influence on face perception. Our participants were instructed to press a button when presented with an inverted face, and it would be interesting to investigate the responses to face-like objects without such priming for faces.

In the set of experimental paradigms, we first performed OBJECTS, in order to avoid both circularity [83] and habituation effects with regard to the neutral Face stimuli. EMOTIONS was performed afterward, which may decrease amplitudes due to habituation, but will be less problematic since it was only used to identify and construct the labels for the FFA.

We performed cluster-based permutation testing to inductively investigate the entire time-series while correcting for multiple observations across time-points. We also performed ANOVA to test a priori hypotheses regarding individual components, resting on previous studies on the topic. Since we had two different statistical outcomes for the same data, we presented both as a “light” version of a multiverse analysis [84] in order to triangulate the significant differences using different approaches. Briefly, permutation testing only identified an FDR-corrected significant difference between faces and objects in the control group. However, there were pre-corrected and borderline significant differences in the same comparisons that were significant in the ANOVA. This indicates that the analyses identify the same findings, but the permutation tests are underpowered to detect differences within narrowly constrained time-windows due to their analysis of entire waveforms. For example, mixed ANOVA indicated a significant difference for M170 amplitude in the autism group for objects compared with faces and face-like objects. Although permutation testing indicated non-significant, but borderline, differences (*p* = 0.053–0.067) across the waveform, it showed a large subtraction difference and effect size corresponding to the M170 component, illustrating the difference in statistical power. We used the permutation tests and multiple subplots in Figs. 5, 6, 7 and 8 as hypothesis driving analyses for future studies, and to incur an additional level of qualitative analysis beyond our narrowly defined time-windows.

One must be wary about analyzing the output for the cluster-based permutation tests. It is not possible to draw clear conclusions about the absolute time-point when significant differences occur. Identifying significant time-windows, and running additional cluster-based permutation tests on those shorter time-windows induces circularity in the analysis [85]. At most one can indicate rough time-windows when significant clusters occur, which can act as hypothesis generating for future studies to specifically investigate those time windows. Besides the permutation tests, we did qualitative analyses that took into consideration several quantitative measures (such as ERFs with confidence intervals, subtraction plots, effect sizes, and within-subject subtractions), as well as t-values over time (to identify the largest clusters within the permutation testing). This is necessary to paint a complete picture, as individual measures may be inadequate, or even yield false positives.

## Conclusions

This study aimed to investigate the event-related fields within the FFA using magnetoencephalography when individuals with and without autism were presented with faces, face-like objects, and objects. Across groups we found that the early (M130/M170) and late components differentiated face-stimuli based on first- and second-order relational properties respectively. There was no between-group difference in the way in which individual stimuli were processed, meaning that face detection is probably intact in individuals with autism of normal cognitive ability. However, within-group comparisons showed less pronounced differences between stimuli for the autism group at later latencies. Considering the increasing involvement of higher cognitive processes with increasing post-stimulus time, this may be taken to argue in favor of such processes being disproportionally affected in autism within the hierarchy of visual processing of a socially relevant stimulus. The finding was not associated with either IQ or autistic-like traits. Since higher cognitive processes are often impaired in both autism and other neurodevelopmental disorders, the result may relate to clinical status (as a nonspecific risk factor), rather than to the autistic behavioral phenotype per se. Future studies should include higher post-stimulus latencies, other neurodevelopmental disorders, and stratify for cognitive ability.

## Supplementary Information

Supplementary Material 1.

## Data Availability

The datasets used and/or analyzed during the current study are not publicly available due to privacy reasons but are available from the corresponding author on reasonable request.

## Abbreviations

ANOVA: Analyses of Variance
dSPM: Dynamic Statistical Parameter Maps
F: Face
FLO: Face-Like Object
O: Object
FFA: Fusiform Face Area
ERF: Event-Related Field
MEG: Magnetoencephalography
MRI: Magnetic Resonance Imaging
IQ: Intelligence Quotient
