# Supplementary material for: Differential late-stage face processing in autism: a magnetoencephalographic study of fusiform gyrus activation
Source: BMC Psychiatry. 2024 Dec 18;24:900. doi: 10.1186/s12888-024-06400-z (PMC11653706; doi:10.1186/s12888-024-06400-z)
Supplement: Supplementary file 1 — Supplementary Material 1. [file 12888_2024_6400_MOESM1_ESM.pdf]

Supplementary material

MEG participant preparations

Participants were instructed not use any hair related products (such as hair gel) on the day of scanning and were changed into non-magnetic MEG-compatible clothes for the scan. To allow for co-registration between MEG and MRI data, the head shape and position of three fiducial points (nasion, left and right auricular points) and four head position indicator-coils (mastoids and superolaterally on the forehead) were digitized using a Fastrak system (Polhemus Inc., Colchester, VT) integrated with the Neuromag system. The coils were digitized once before and once after the digitization of the head shape and fiducial points, and an in-house developed script was used to identify any movement between the two digitizations to assure that movement of the coils had not occurred, in which case the digitization process was repeated. Position of head origin within the helmet was localized before each paradigm, and following breaks within paradigms, and was continuously monitored during the MEG session in order to compensate for head motion.

The electrocardiogram (ECG) – used to identify heart beats for artifact removal – was recorded using two electrodes attached below the left and right collarbones. Horizontal and vertical electrooculography (EOG) was recorded using electrodes placed at the outer canthi of the eyes and above/below the left eye respectively, and was later used to identify saccades and eye blinks for artifact removal. The ground electrode was placed at the back of the neck. A MEG-compatible eye-tracking device (SR Research Eyelink 1000) was used to record gaze direction, and to ensure the stimuli were viewed as instructed.

MEG scanning

MEG, ECG, and EOG signals were sampled at 1000 Hz. The entire MEG recording consisted of the following experimental paradigms: resting state (5 min), faces, face-like objects and objects (OBJECTS; 30 min), moving circular gratings (8 min), faces with different emotions (EMOTIONS; 20 min), moving circular gratings (8 min), and resting state (5 min). Only the paradigms involving face-stimuli were used in the present study, and the others will not be presented further. An empty room recording for 5 min was performed before each participant entered the room, which was used for estimating noise levels.

Two experimental paradigms were used for the present study. In the first paradigm (OBJECTS), the participants were presented with 120 black and white neutral faces (F), 240 face-like pareidolic objects (FLO), 120 common objects (O), and 80 inverted neutral faces in random order. In the second paradigm (EMOTIONS), which was used to localize the FFA, the participants were presented with black and white images of faces expressing different emotions (100 neutral faces, 100 happy faces, 100 angry faces, 60 inverted neutral faces).

For both paradigms, to maintain vigilance, the participants were tasked with pressing a button with their dominant hand as soon as possible whenever an inverted face appeared. The stimuli were presented on a back-projection screen placed 150 cm in front of the participant. The order of the stimuli was randomized. They were presented for 800 ms with an inter-stimulus period of 2600 to 3400 ms consisting of a blank screen with a fixation cross. The stimuli were presented inside a circle with a width of 480 pixels to preserve a constant visual angle. The brightness of the screen and inside the magnetically shielded room were kept constant within and between participants.

There was a one-way auditory connection between the magnetically shielded room and laboratory, and the participants were video-monitored throughout the MEG recording session. Before and after each experimental paradigm, the participants were instructed to grade their sleepiness from 1 to 5 on a Likert scale. When a high sleepiness score was recorded, or if the researchers noted that the participant was visibly sleepy, lacking attention, or moving excessively, a break was made. If the participant was visibly tired or had moved excessively compared with the starting position, the recording was paused. At the start of each recording, and after each break, the position of the head within the helmet was measured to ensure that it did not change more than 5 mm in relation to the original position.

MEG data preprocessing

Elekta proprietary MaxFilter was applied to compensate for head movements, and to eliminate environmental magnetic noise and the signal from active shielding using temporal Signal-Space Separation. Bad channels and segments of data with excessive noise or movement were excluded based on visual inspection. The data was then band-pass filtered between 1 Hz and 40 Hz before using Independent Component Analysis for identification of biological artifacts (eye blinks and cardiac activity, using ECG- and EOG- channels respectively). Prior to Independent Component Analysis, the rank of the MEG data was reduced to 30 (or 40 if

artifacts could be identified across fewer components, resulting in lower rates of exclusion) using Principal Component Analysis (i.e. 30 PCA components). The independent components representing ECG- and EOG-related artifacts were identified using the automatic MNE-python module for ICA artifact detection, and visually inspected to identify their artifact nature and then removed. Resulting ERFs for all sensors were visually inspected to identify the optimal trade-off between the number of removed components and artifact removal. This excluded on average 10.2% ± 2.4% and 10.6% ± 2.6% components from the autism and control groups respectively.

The Independent Component Analysis-correction was then applied to the raw data and a band-pass filter (a non-causal overlap-add FIR filter with zero phase) between 1 Hz and 40 Hz was applied to the corrected data. Bad channels and segments of noisy data were manually annotated and excluded from analysis. Data were then epoched around the stimulus onset (from 200 ms before to 800 ms after) and baseline corrected using the 200 ms pre-stimulus interval. Epochs with average amplitudes exceeding 5e-12 fT for magnetometers and 4e-10 fT for gradiometers were automatically excluded. For the OBJECTS-paradigm, three individuals were excluded because of excessive artifacts or noise, and the average number of excluded epochs (out of 480 presentations) in the rest of the subjects were 5.6 ± 7.5 and 4.9 ± 6.5 for the autism and control groups respectively (with no significant difference between the groups, p = .74).

The epochs were then averaged for each individual to generate ERFs. For the EMOTIONS-paradigm a grand average was generated for each individual using all upright stimuli (neutral, happy, and angry faces). For the OBJECTS-paradigm, the ERFs were averaged for each stimulus and group (used for between and within group comparisons), and for the stimuli across all participants. Inverted faces were only used to maintain and assess vigilance, and therefore excluded from further analysis in both datasets.

Fusiform face area localization

FFA-labels from previous studies using functional MRI with neutral faces were available for seven participants, and were used to confirm the location of the FFAs in the MEG dataset. Since the same source space distribution was used for all participants, the number of vertices within the outer bounds of the label was used to estimate the size of each individual's FFA. The autism and control groups had label sizes of 309 ± 116 and 337 ± 142 vertices respectively.

There was no significant group difference (p = .48), excluding the possibility that differences in label sizes contribute to group ERF differences.

MEG data analysis

Spatial permutation on source space activation

Following exclusion of the labels for the medial brain, the MEG data for each individual was mapped onto the entire cortical surface. Each participant's cortical pattern of gyri and sulci, and their source localized activation, were morphed onto a common structural template (fsaverage), allowing for comparisons to be made between participants. The source localized activation was downsampled to 50 Hz, and shortened to include a time window between 50 and 200 ms, in order to speed up calculations. An adjacency matrix was computed for the dipole distribution.

Statistical analyses

False discovery rate correction

We corrected for multiple observations to limit the risk of type I error associated with performing multiple analyses. We performed FDR-correction according to the number of observations within independent sets of analyses, such as between- and within-group comparisons. Performing FDR-correction for the total number of observations would be too conservative, due to the covariance of dependent datasets. For that reason, we performed sequential Holm-Bonferroni correction (Holm, 1979) on the alpha-level within each family of analyses: mixed ANOVA for 5 dependent variables, one-way ANOVA for ten (2 x 5) comparisons, and between- and within-group permutation testing for six (2 x 3) comparisons each.

The Holm-Bonferroni method is equally stringent with regard to type I errors, but is more powerful with regard to type II errors than Bonferroni, and involves comparing each p-value with that of the alpha-level associated with the rank of the significant p-values given by the Holm-Bonferroni formula:

Corrected alpha = target alpha / (number of observations – rank of p-value + 1)

ANOVA

Since the majority of the dependent variables correlated less than .1, rather than performing a multivariate ANOVA, we performed a mixed ANOVA for each dependent variable and FDR-corrected for an equal number of tests.

Equality of variances and sphericity were tested. Welch test was performed when Levene's test was significant, and Greenhouse-Geisser corrections were performed when Mauchly's test of sphericity was significant.

Cluster-based permutation testing

Since time-series data contains thousands of datapoints and potential comparisons, we also employed non-parametric cluster-based permutation testing that corrects for multiple observations across the ERFs. Cluster-based permutation tests the entire ERF, and as such includes the discrete time-points tested in the mixed ANOVA. However, its statistical power is slightly lower for each individual time-point and doesn't allow for testing of time-windows within the time-window of analysis. Since we had a priori hypotheses regarding the M130 and M170 components, based on previous studies in the literature, we also performed ANOVA on those time-points.

We performed between-group comparisons for Group (autism vs control for three stimuli) and Stimulus (three stimulus comparisons across all individuals). In order to investigate within-group differences, we also compared the stimuli within each group individually, with three stimulus comparisons for each group.

Significance across time

By extracting the T-values from each permutation, one can illustrate the significance across time. Rather than just identifying the size of the largest T-value cluster above a threshold, one can use the matrix of randomized permutations (T-values for all permutations across each time-point from -100 to 700 ms) and compare the T-value of the actual data against the distribution of permutated T-values. This allows for visualization and qualitative assessment of each time-point within the confines of the permutation testing, and thus provide an illustration of the temporal extent of the largest clusters. We present this as a hypothesis generating mechanism for future studies that can make use of a-priori assumptions about the ERFs (Sassenhagen & Draschkow, 2019).

Results of across-group stimulus comparisons using cluster-based permutation testing

Faces vs objects

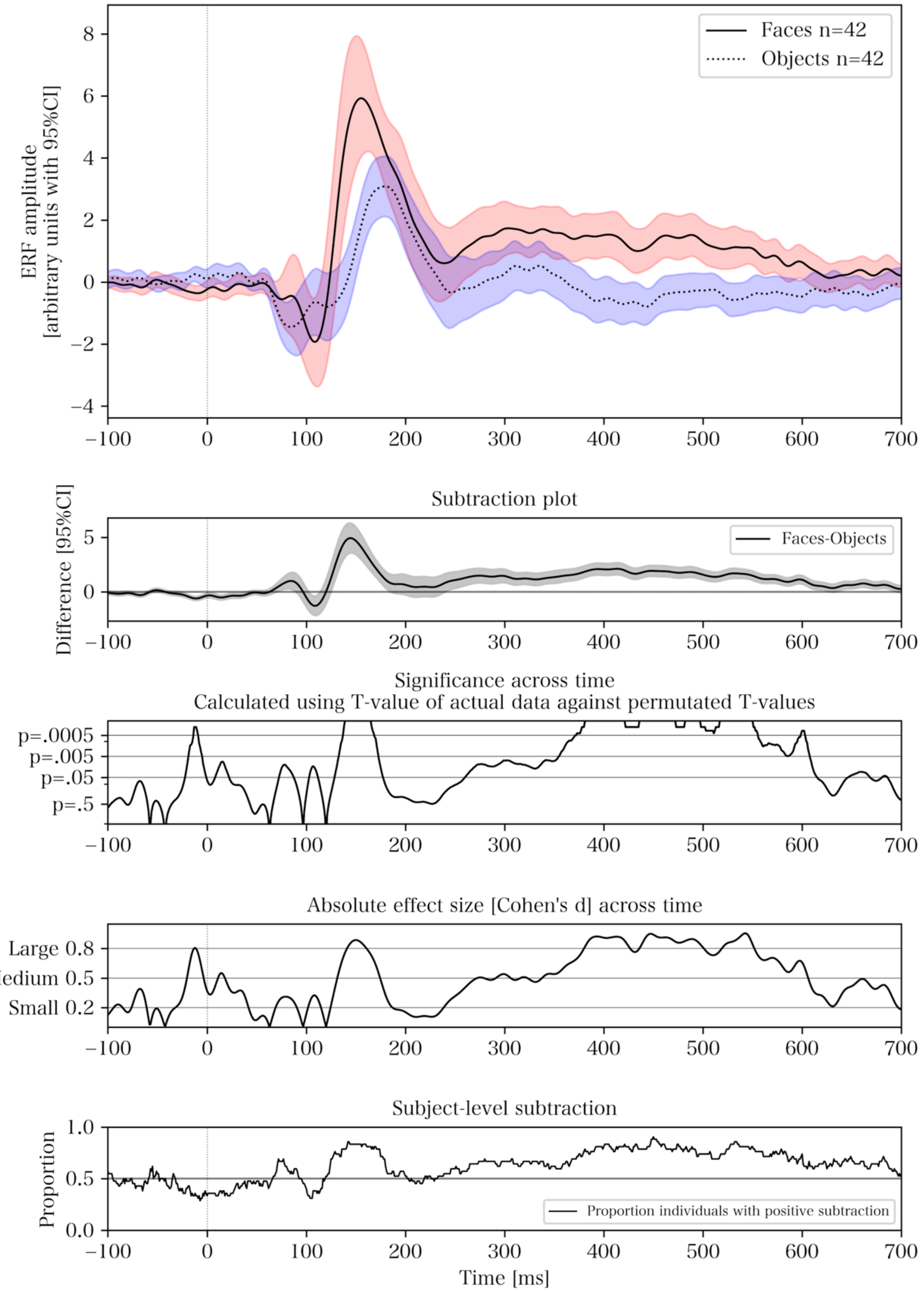

Figure S1. Across-group pairwise comparison between faces and objects (p = .0001) using cluster-based permutation test for event-related fields with 10000 permutations.

Face-like objects versus objects

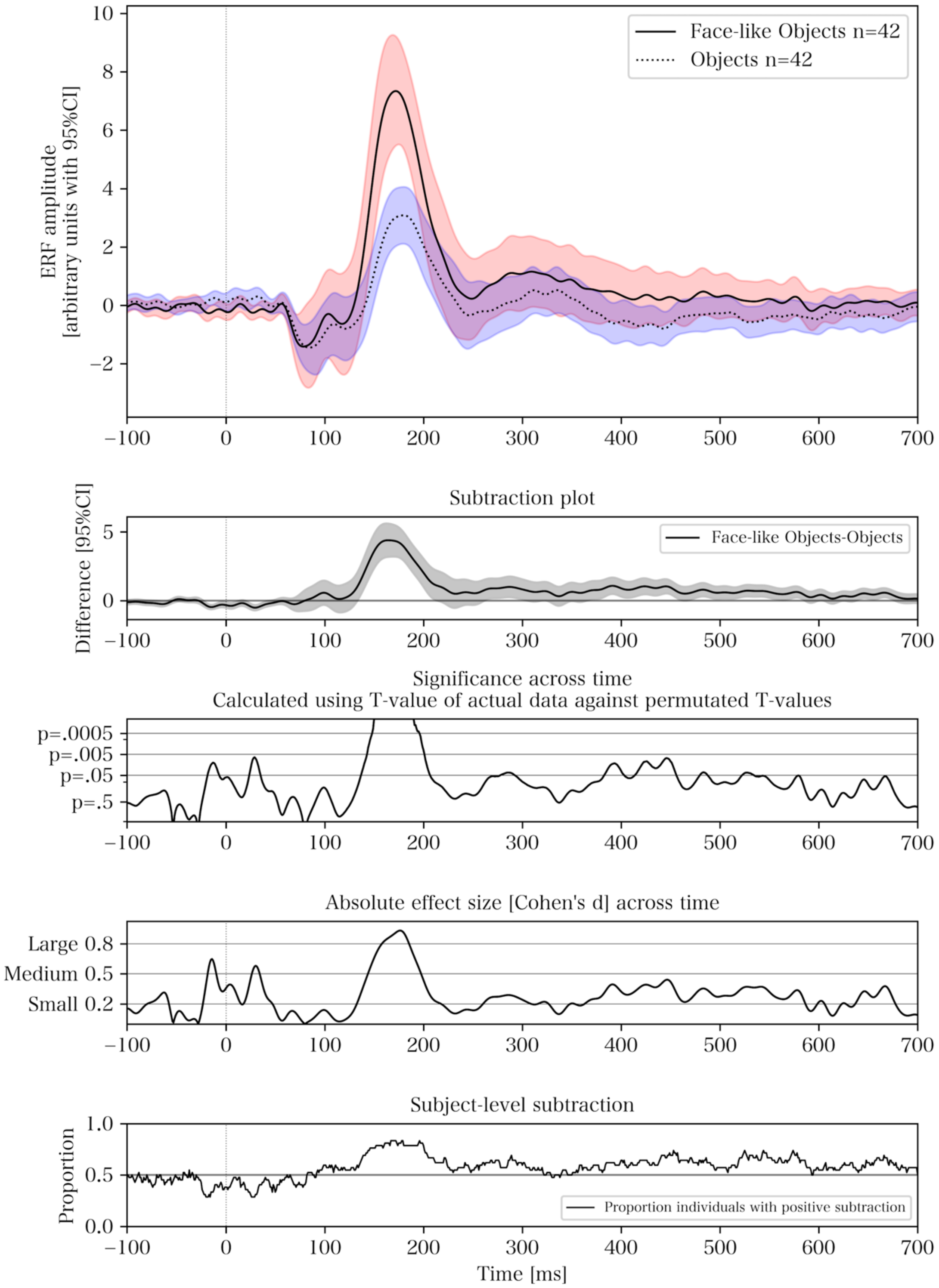

Figure S2. Across-group pairwise comparison between face-like objects and objects (p = .0082) using cluster-based permutation test for event-related fields with 10000 permutations.

Faces versus face-like objects

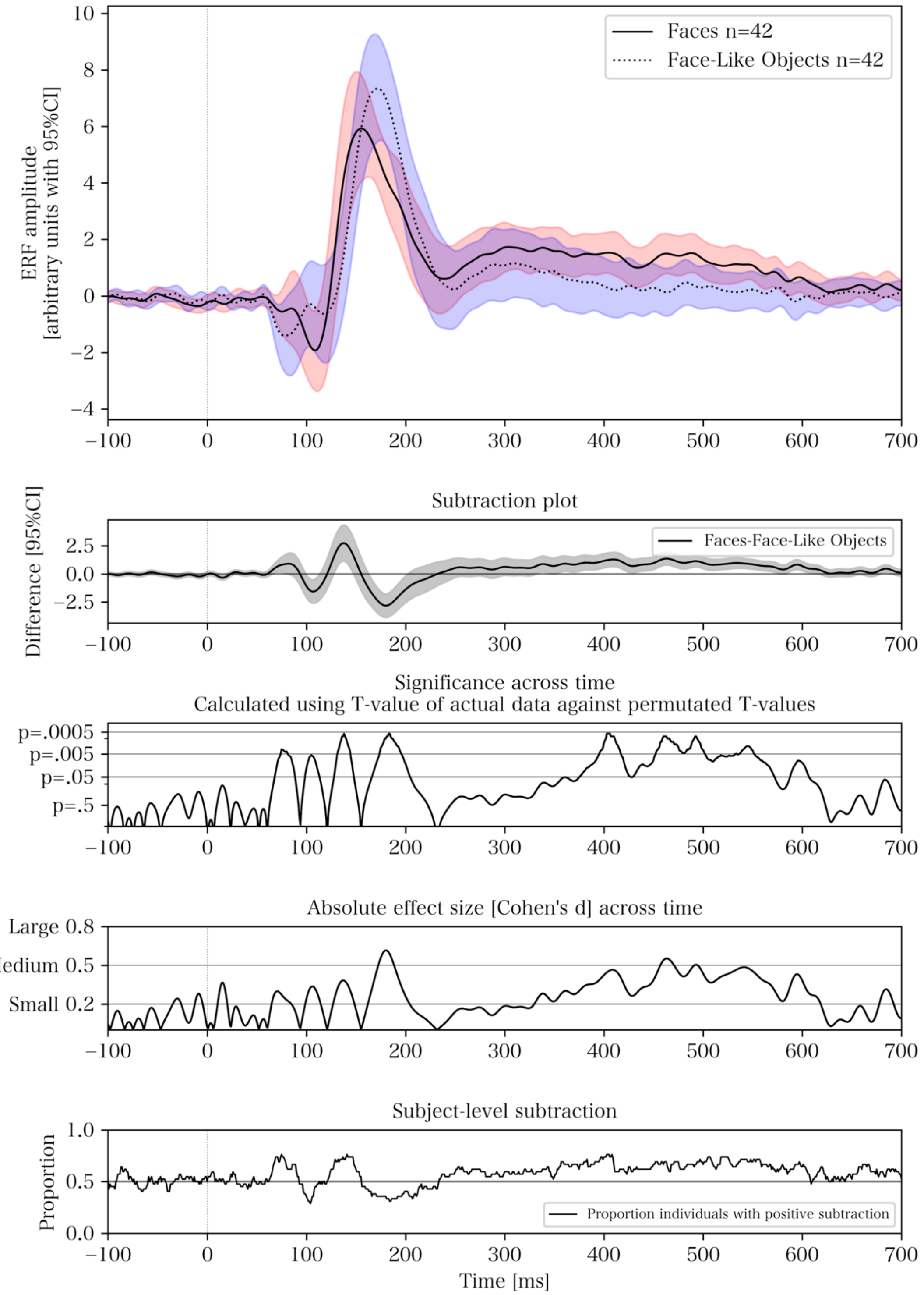

Figure S3. Across-group pairwise comparison between faces and face-like objects (p = .01) using cluster-based permutation test for event-related fields with 5000 permutations.

References

- Holm, S. (1979). *A simple sequential rejective multiple test procedure*. Scand J Stat, 6:65-70.
- Sassenhagen, J. and D. Draschkow. (2019). *Cluster-based permutation tests of MEG/EEG data do not establish significance of effect latency or location*. Psychophysiolog, 56(6): p. e13335.
